# An unsupervised learning approach for tracking mice in an enclosed area

**DOI:** 10.1186/s12859-017-1681-1

**Published:** 2017-05-25

**Authors:** Jakob Unger, Mike Mansour, Marcin Kopaczka, Nina Gronloh, Marc Spehr, Dorit Merhof

**Affiliations:** 1 Institute of Imaging and Computer Vision, RWTH Aachen University, Kopernikusstr. 16, Aachen, 52056 Germany; 2 Department of Chemosensation, Institute of Biology II, RWTH Aachen University, Worringer Weg 3, Aachen, 52074 Germany

**Keywords:** Tracking, Mice, Animal behavior, Unsupervised learning, Shape matching, Shape context, Active shape model

## Abstract

**Background:**

In neuroscience research, mouse models are valuable tools to understand the genetic mechanisms that advance evidence-based discovery. In this context, large-scale studies emphasize the need for automated high-throughput systems providing a reproducible behavioral assessment of mutant mice with only a minimum level of manual intervention. Basic element of such systems is a robust tracking algorithm. However, common tracking algorithms are either limited by too specific model assumptions or have to be trained in an elaborate preprocessing step, which drastically limits their applicability for behavioral analysis.

**Results:**

We present an unsupervised learning procedure that is basically built as a two-stage process to track mice in an enclosed area using shape matching and deformable segmentation models. The system is validated by comparing the tracking results with previously manually labeled landmarks in three setups with different environment, contrast and lighting conditions. Furthermore, we demonstrate that the system is able to automatically detect non-social and social behavior of interacting mice. The system demonstrates a high level of tracking accuracy and clearly outperforms the MiceProfiler, a recently proposed tracking software, which serves as benchmark for our experiments.

**Conclusions:**

The proposed method shows promising potential to automate behavioral screening of mice and other animals. Therefore, it could substantially increase the experimental throughput in behavioral assessment automation.

## Background

Targeted mutations in mice have been successfully employed for understanding gene function, testing hypotheses and developing treatments for human genetic disorders [1–3]. In particular, mouse models are used to uncover disease mechanisms underlying neurocognitive disorders such as autism or schizophrenia. By modifying candidate genes that cause specific mental disorders in mice, correlations between targeted mutations and behavioral phenotypes are identified making mouse models a valuable tool for neuroscientists. Measures of social interactions and behavior in mouse models are crucial read-outs. However, manual documentation of behavioral complexity in mice remains highly subjective and may not provide reproducible results. Furthermore, the frame-by-frame assessment of long video tape recordings is time-consuming and still constitutes a bottleneck in large-scale studies. In this respect, high-throughput behavioral screening systems can overcome the aforementioned weaknesses of manual assessments.

From a technical point of view, automated simultaneous tracking of two or more individuals and online classification of their interactions and behavior are challenging tasks. While tracking is straightforward when all individuals are spatially separated, task automation is complicated when animals directly interact. In this case, additional knowledge about shape or texture has to be taken into account to separate individual shapes. A straightforward method to keep track of individuals during interactions is to label each subject with a unique marker, i.e., by bleaching [4], color [5] or RFID technology [6, 7]. Labeling, however, has a direct impact on the environment and frequently provides a sensory (i.e., olfactory and / or visual) stimulus and, thus, it may influence an individual’s social behavior.

When markers are omitted, automatic assessment of social interaction is challenging. Several approaches have been proposed to tackle this problem. Identification of individuals has been addressed by ellipse fitting [8], watershed segmentation [9] or particle filters [10, 11]. In some of these studies, camera images are complemented by additional sensor data such as infrared [9] or depth sensing [8]. Generally, using complementary modalities enhances tracking reliability but involves additional hardware and demands a careful calibration. All these approaches, however, do not incorporate prior knowledge about the anatomy and motion patterns of the individuals to be tracked.

Model-based tracking systems have been designed for different animals, specifically drosophila [12], bees [13] and mice [14]. In order to provide a reliable tracking routine, the anatomy of the animals is modeled by connected rigid primitives representing the head, thorax, abdomen or wing. The model parameters allow to document complex motion patterns and furthermore provide information about the orientation and distance for each individual body part, which in turn allows more complex behavioral state and body pose categorizations. Thereby, the degree of generalization constitutes a crucial trade-off between the time needed to adapt the model to a specific scenario and the performance achieved in specific cases.

In this paper, we pursue a different strategy by automatically building a model during runtime that facilitates tracking when individuals interact closely. In the first step, shape information of the individuals is learned and documented in a catalog as long as they are spatially separated. The second step involves training of an active shape model (ASM) using the previously defined shape catalog to separate the individuals when they are in close proximity. The benefit of this procedure is twofold: first, the shape information gathered in the first step constitutes a-priori knowledge that helps to keep track of the individuals in challenging conditions and, secondly, the ASM eigenvalues provide additional information about behavioral states. Therefore, the proposed method provides features to identify specific conditions and social interactions. Moreover, all manual interaction that is required before the tracking process (the user has to determine head, nose and ear landmarks only once on a reference shape) is completed within a few seconds.

The proposed method is validated by comparing manual annotations with estimated position of head and tail landmarks as well as viewing directions of pairs of mice (male/male, female/female, male/female) interacting in three different environments. From the set of tracking parameters and the eigenvalue data, social and non-social interactions are classified. The approach presented shows wide agreement between manual labeling and automatic classification. This allows for a substantial increase of experimental throughput in behavioral assessment automation with only a minimum level of user intervention.

## Methods

### Animals

All animal procedures were approved by local authorities (AZ 39.3-60.06.04) and in compliance with European Union legislation (Directive 2010/63/EU) and recommendations by the Federation of European Laboratory Animal Science Associations (FELASA). C57BL/6 mice (Charles River Laboratories, Sulzfeld, Germany) were housed in groups of both sexes (RT; 12:12 h light-dark cycle; food and water available ad libitum).

### Experimental setup

**Fig. 1:**
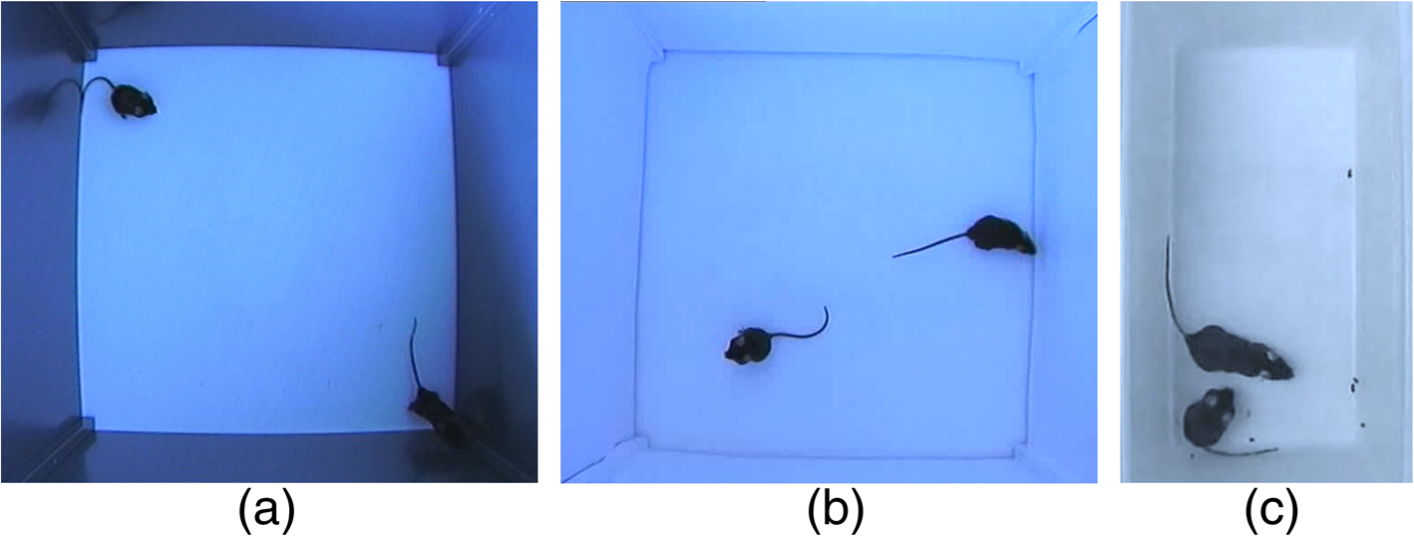
Three different arena setups. **a** First setup: two female mice in an open arena with slightly reflecting walls and reduced contrast. **b** Second setup: walls are covered with *white paper* providing enhanced contrast and reduced reflections. **c** Third setup: Pairs of mice male-male/male-female in a cage

The tracking and phenotyping experiments were carried out in a rectangular open field arena with a size of 45 cm × 45 cm or a standard cage with a size of 16.5 cm × 32.5 cm. The animals were recorded with a Panasonic WV-CP480 camera providing a spatial resolution of 768×494 pixels at 25 frames per second from a top-view. First, the open field was prepared in two different setups where two female C57BL/6 mice were placed. In a first setup the arena was equipped with wooden walls painted in a dark blue with moderate reflectance providing a poor contrast to the black mice to simulate challenging tracking conditions (Fig. 1 a). Second, the walls were covered with white paper which considerably reduced reflectance and enhanced contrast conditions (Fig. 1 b). The second setup provides much better preconditions for automated tracking and behavioral phenotyping. However, the white background and altered illumination conditions may provoke considerably different patterns of behavior and stress [15, 16]. Consequently, an automated assessment should ideally cope with both scenarios. In a third setup, mice were placed in a cage (Fig. 1 c) and the scene was recorded with the same camera. A male-male and male-female combination was considered. Especially the male-female setup provides a higher variability of close interactions posing a particular challenge for the tracking system.

### Video data and manual annotation

In order to validate tracking and behavioral phenotyping performance, two videos, each with a length of 20 min and two videos, each with a length of 10 min were recorded and processed: video 1 (V1) using the first setup, video 2 (V2) with optimized contrast and reflectance conditions, video 3 (V3) with two male mice in a cage and video 4 (V4) with a male and a female mouse in a cage. The ground truth of position and orientation of both mice was manually labeled for each video. The manual assignment includes the nose tip, tail base and the viewing direction. Furthermore, grooming and mating behavior was documented (see “Social behavior classification” section). The manual assessment also included keeping the identity of each mouse to assess the tracker’s ability to assign the correct identities to both animals during interactions. To reduce the effort of labeling, every fifth frame was labeled in each video, resulting in a total number of 18,000 manually labeled video frames. Annotations were made with a Matlab program specially designed for labeling nose, tail, ears and the viewing direction.

### Social behavior classification

**Fig. 2:**
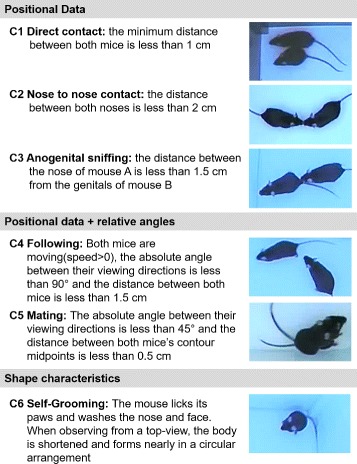
Social (C1-C5) and non-social (C6) conditions. Conditions C1-C3 are determined by positional data settings, C4 and C5 additionally incorporates relative angles and C6 is characterized by the outer shape of the mouse body

Based on several previous studies, we adopted a list of behaviors and social interactions [14, 17, 18] that are based on positional data, viewing direction and shape characteristics (Fig. 2). Social interactions (C1-C4) are identified according to the tracking results as defined in [14]. This categorization defining interactions have shown good agreement with human ratings [14]. Mating behavior (C5) was evaluated for video V4. The first three conditions are based on positional information whereas categories 4 and 5 also include relative angles between the viewing directions. Self-grooming (C6) was found to be evident for mouse models in the context of autism [18] and can be identified according to the outer mouse segmentation when observed from a top-view.

### Validation

To compare the performance, the MiceProfiler tracking software [14] served as benchmark for the proposed method. The MiceProfiler is a sophisticated software system based on physics engines [19, 20] that has been evaluated comprehensively [14]. Tracking accuracy of the proposed method was validated by computing the Euclidean distances

1 $$ d_{f}^{Nose,\{USM,MP\}} = \left\| P_{f}^{Nose,GT}-P_{f}^{Nose,\{USM,MP\}}\right\|  $$

and

2 $$ d_{f}^{Tail,\{USM,MP\}} = \left\| P_{f}^{Tail,GT}-P_{f}^{Tail,\{USM,MP\}}\right\|  $$

between the key landmarks nose $P_{f}^{Nose}$ and tail base $P_{f}^{Tail}$ as estimated by the proposed unsupervised learning method (USM) or the Mice Profiler (MP) and the corresponding manually labeled ground truth (GT) where *f* denotes the f-*th* frame. Analogously, the angular deviation

3 $$ \Delta \varphi_{f}^{\{USM,MP\}} = \left\|\varphi_{f}^{GT}-\varphi_{f}^{\{USM,MP\}}\right\|  $$

between labeled and estimated viewing direction was evaluated. Based on the tracking results, the interactions 1–5 (Fig. 2) were automatically identified according to positional data and viewing angles provided by both tracking algorithms. For the self-grooming condition C6, additional shape related data has to be considered. In the current implementation, the Mice Profiler system does not incorporate this information. The automated identification of C6 is therefore evaluated only for the proposed method.

**Fig. 3:**
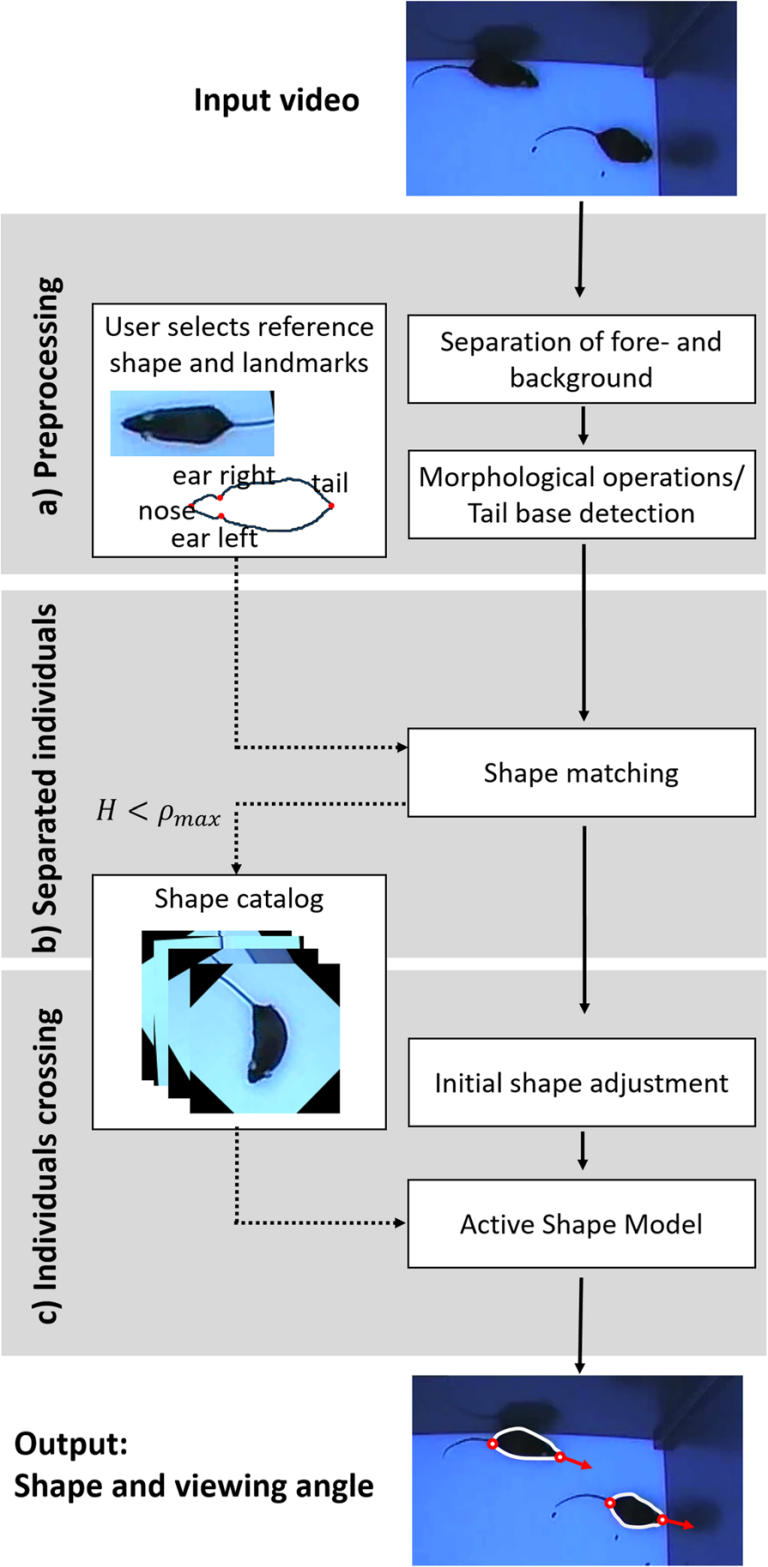
Processing pipeline of the tracking routine. The method consists of three subsequent blocks: **a** Preprocessing, **b** Separated individuals: Segmentation and shape learning **c** Individuals crossing: Using deformable models to segment individuals during interactions

Figure 3 summarizes the three consecutive steps of the proposed method. After the preprocessing steps (A) the frames are divided into two categories: both individuals are separated (B) or in direct contact (C). If they are spatially separated, they can be easily distinguished and segmented. In this case, both mice segmentations are matched to a reference shape that has been previously selected from an arbitrary frame and annotated by the user. The matching results provide information about the orientation and viewing angles and furthermore, they are stored in a shape catalog describing the variations of their shapes. Subsequently, an ASM is built on the basis of the previously created shape catalog in order to separate the individuals during direct interactions. The procedure is explained in detail in the following sections.

#### Preprocessing: background separation

A static background is presumed for the proposed algorithm. The focus is put on the individuals actively moving within the scene whereas the background is removed. First, the frames are converted to grayscale and temporal illumination inhomogeneities are removed for each frame separately by dividing each pixel intensity by the mean image intensity and scaling back to an adequate intensity range. The static background is eliminated by taking the pixel-wise median over time and subtracting it from each frame. Note that background subtraction is a common way to separate objects from a scene [12, 14, 21, 22] and was demonstrated to work well as long as the background is static and the contrast is good enough [12, 14, 21]. The automatic thresholding worked well for all the videos that we tested. However, if the automatic setting fails for any reasons, it can be adapted manually.

##### Blob extraction

The shapes acting in the foreground, in the following referred to as blob objects, correspond to the individuals moving. To obtain a precise delineation of these blobs, a simple thresholding routine [23] is applied. Remaining artifacts can be removed by defining a minimum blob size *b*_*min*_ which can be set arbitrarily by the user before the tracking routine is initiated.

##### Morphological operations

For the following shape extraction and learning routines (step B of the pipeline), the tails of the animals are removed. The rationale is twofold: Firstly, the tails are frequently disappearing in the binary segmentation [9]. The shape matching algorithm thus may fail when matching animal shapes with and without tail. The second point is that the relative orientation of body and tail are rather uncorrelated. Shape variances to be learned for the active shape model are thus getting much more complex for shapes where the tail is included.

**Fig. 4:**
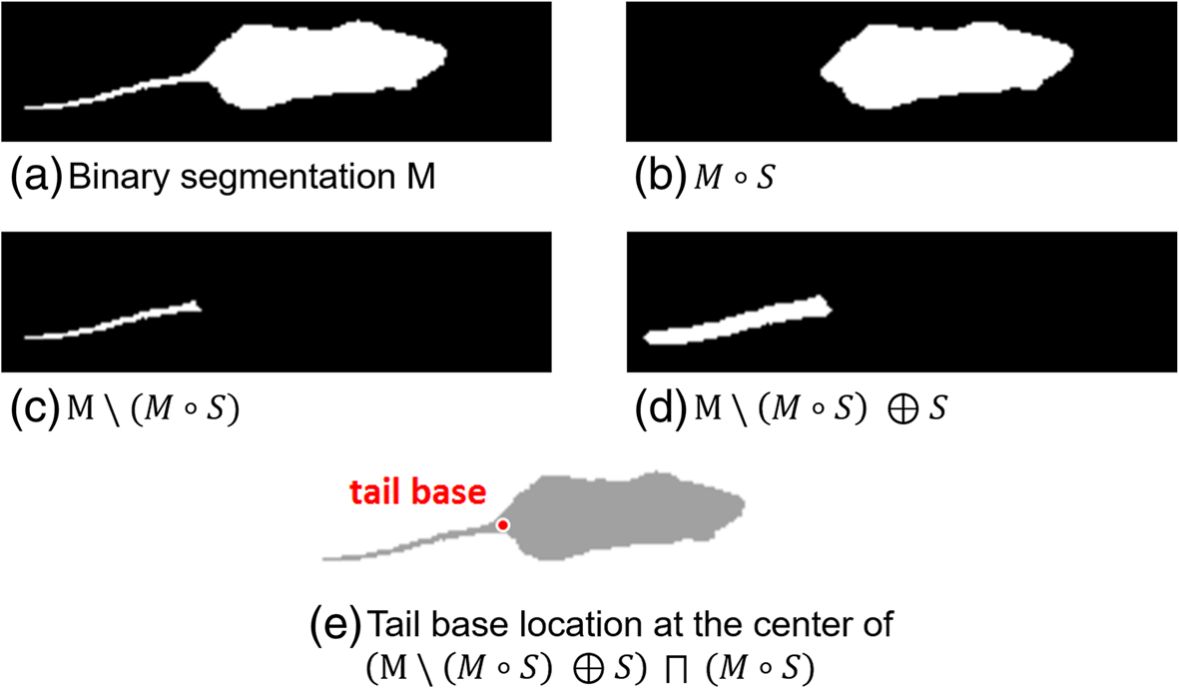
Tail base localization. A series of morphological operations (**a**)–(**e**) is applied to localize the tail base. It is obtained from the center of the intersection of the body (**b**) and the dilated tail (**d**)

As nose and tail points are easily switched when analyzing mice shapes, detecting the tail position provides additional information as it indicates the orientation of the segmented body.It is thus employed to enhance the robustness of orientation estimation during shape matching (see “Shape matching” section). A series of morphological operations is performed on the binary segmentation *M* to localize the tail base (Fig. 4). First, the tail is extracted by subtracting the result of a morphological opening from the original segmentation (Fig. 4 c). Finally, the tail base is given by the center of the intersection of the dilated tail (Fig. 4 d) and the body (Fig. 4 b). The structural element *S* is chosen as open disc of radius *r*_*S*_. Note that the radius *r*_*S*_ depends on the diameter of the tail and should be chosen accordingly.

#### Separated individuals: shape learning process

The preprocessing step yields blob objects where each blob may contain one or two individuals. In a next step, a catalog of shapes is built. The first step in catalog building is the identification of blobs where the individuals are entirely separated and do not cross or touch. The set of video frames where both individuals are separated is denoted with *F*_*S*_ and the set comprising the remaining frames analogously with *F*_*C*_.

##### Initializing the learning process

Initially, the user selects a representative separated mouse shape (preferably in a straight alignment) from an arbitrary frame that is to be tracked. The boundary

4 $$ \mathbf{x}=\left(x_{1}, y_{1}, \ldots x_{n}, y_{n} \right)^{T}  $$

obtained from the corresponding blob object is referred to as reference shape. Subsequently, the user marks meaningful boundary landmarks, i.e. head, tail and ear positions (Fig. 3). In a second step, all boundaries extracted from *F*_*S*_ are mapped to the reference shape using the shape context matching and the inner-distance as proposed by Ling and Jacobs [24] and as described in the next “Shape matching” section. As nose and tail base of the matching may be easily switched, the matching is aligned to the tail base that has been localized using the previously described morphological operations (see “Preprocessing: background separation” section). If the tail base cannot be localized, i.e. through occlusions, then the orientation is aligned according to the previous frame.

##### Shape matching

Belongie et al. [25] proposed a shape matching procedure based on a log-polar histrogram. For each contour point *p*_*i*_=(*x*_*i*_,*y*_*i*_)^*T*^, the distribution of the remaining contour points is represented by the log-polar histogram

5 $$ h_{i}(k)=\# \left\{ q \neq p_{i} : (q-p_{i}) \in bin(k) \right\},  $$

where *b* *i* *n*(*k*) denotes the *k*-th bin of the log-polar space. The costs of matching two points *p*_*i*_ and *p*_*j*_ are given by the *χ* ^2^ test

6 $$ C(p_{i},p_{j}) = \frac{1}{2} \sum\limits_{k=1}^{n} \frac{\left[h_{i}(k)-h_{j}(k)\right]^{2}}{h_{i}(k)+h_{j}(k)}.  $$

Note that due to the logarithmic distance scaling, the cost function is more sensitive to nearby contour properties. Minimizing the total costs

7 $$  H(\pi)=\sum\limits_{i} C(p_{i},q_{\pi (i)}),  $$

where *π* is a permutation, finally yields an optimal bipartite graph matching providing the desired correspondences for the graph matching. A detailed description of the algorithm and a corresponding implementation, is available in Belongie et al. [25].

However, the shape context matching relies on Euclidean distance measures. Anatomical conditions of animals, such as the flexibility of the spine, allow for a high variance of shape delineations. A straightforward extension which is less sensitive to articulations has been proposed by Ling and Jacobs [24]. There, the Euclidean distance is replaced by the inner-distance, defined as the shortest path between landmark points within a shape silhouette [24]. The relative angle between two points is replaced by the inner-angle, which is defined as the angle between the tangent at the starting point *p* and the initial direction of the shortest path [24]. These modifications allow for a better matching performance for animal shape silhouettes and are therefore employed for the proposed shape learning process. Particularly, the inner-distance matching proved to be very successful for tracking mice from a top-view [26].

##### Shape catalog

**Fig. 5:**
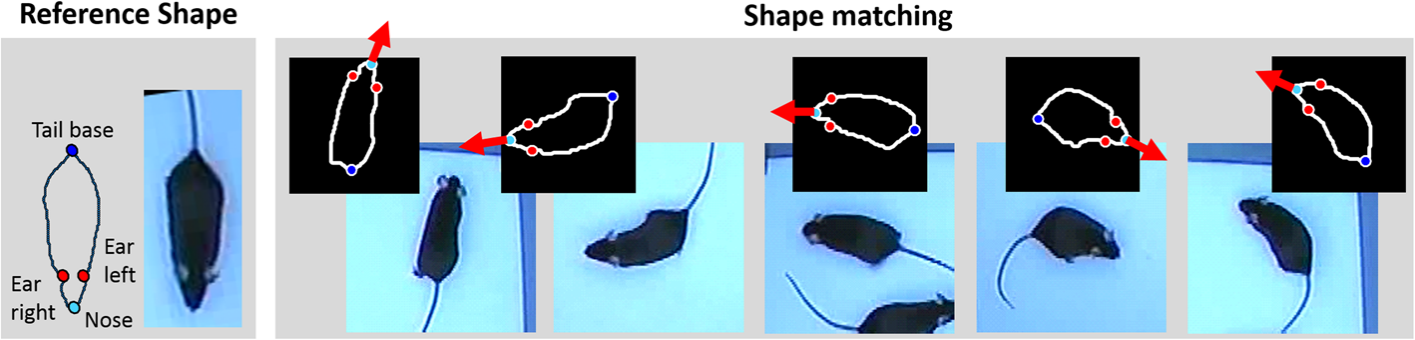
Five matching examples. *Left*: reference shape where tail, nose and both ears are marked, *right*: boundaries matched to the reference shape using the algorithm proposed by Ling and Jacobs [24]. The viewing direction (*red arrows*) is given by the straight line connecting the midpoint between both ears and the nose

As long as both individuals are separated, position and orientation can be directly estimated by matching each blob boundary to the reference shape using the shape context algorithm in combination with the inner-distance measure as described in “Shape matching” section. Point correspondences of head, tail and ear positions are exemplarily shown in Fig. 5 for different mice shapes and the reference shape they are mapped to. The viewing direction is estimated from the line going through the nose point and the midpoint between both ears (red arrows in Fig. 5). In doing so, the estimated viewing direction only depends on the relative head position instead of the whole body alignment as i.e. done by Hong et al. [8].

In a next step, in order to learn variations of animal shapes, a catalog is created. However, it cannot be guaranteed that the matching produces plausible correspondences. As this mismatching tends to have higher matching costs, only shapes and corresponding images in *F*_*S*_, where the total matching costs *H* (Eq. 7) are below a predefined threshold *ρ*_*max*_, are added to the catalog. The threshold level has to be defined by the user before the tracking routine is initiated. High matching costs are often related to slight offsets of the placed landmarks. The threshold therefore constitutes a trade-off between a high variability and plausibility of the shape data and has to be chosen with caution.

Finally, the line connecting head and tail points is aligned to the vertical axis for each shape of the catalog. Eliminating whole-body in-plane rotation from the shape model and working exclusively on vertically aligned shapes allows to drastically reduce the complexity of shape variation while maximizing shape-relevant information in the model’s eigenvectors.

#### Occlusion events: separation of individuals

When two individuals are close together, the segmented blob object covers both individuals. To separate their shapes, an ASM is trained using the shape and image information that has been previously stored in the catalog.

##### Active shape model

**Fig. 6:**
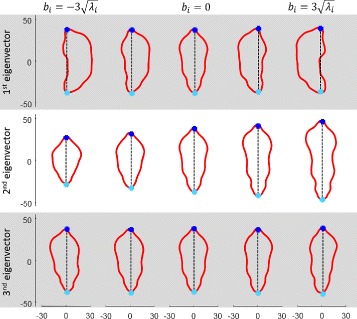
First three eigenvectors of the covariance matrix. The first indicates a left or right turn, the second squash and stretch and the third eigenvalue comprises only slight variations that are difficult to interpret

The ASM was originally proposed by Cootes al. [27] and is closely related to active contour models as introduced by Kass et al. [28]. In contrast to active contour models, the deformation is restricted to shape variations that are previously learned from a training set. From the landmarks **x** of the *s* training images the covariance matrix

8 $$ \mathbf{S_{x}}=\frac{1}{s-1} \sum\limits_{i=1}^{s} \left(\mathbf{x_{i}} - {\bar {\mathbf{x}}} \right) \left(\mathbf{x_{i}} - {\bar {\mathbf{x}}} \right)^{T}  $$

is computed where

9 $$ {\bar {\mathbf{x}}}=\frac{1}{s} \sum\limits_{i=1}^{s} \mathbf{x_{i}}.  $$

is the mean shape of the training set. Consequently, any shape from the training data can be approximated by

10 $$ \mathbf{x} \approx {\bar{\mathbf{x}}} + \mathbf{P} \mathbf{b}  $$

where **P**=(*p*_1_ *p*_2_…*p*_*t*_) denotes the matrix whose columns are given by the eigenvectors **p**_*i*_ and **b**=(*b*_1_,*b*_2_,…,*b*_*t*_) is a vector of weights. Thus, any shape can be approximated by a linear combination *b* of the eigenvectors. As the eigevectors are orthogonal,

11 $$ \mathbf{b}=\mathbf{P^{T}} \left(\mathbf{x}-{\bar{\mathbf{x}}} \right)  $$

allows forming shapes that are closely related to the instants of the training set. To maintain plausibility of the resulting shape, the range of the coefficients *b*_*i*_ is typically restricted to the interval

12 $$ -m \sqrt{\lambda_{i}} \leq b_{i} \leq m \sqrt{\lambda_{i}}.  $$

where *λ*_*i*_ denotes the i −*t* *h* eigenvalue and *m* determines the range of the model parameters. The segmented mouse shapes exhibit a high degree of freedom as their orientation can be arbitrary. A considerable reduction of complexity can be achieved by consistently aligning the shapes in a predefined orientation. Here, the axis connecting tail base and nose points is aligned to the vertical axis where the nose points downwards (see Fig. 3). The first three eigenvectors obtained from the unsupervised learning routine using the vertical alignment are shown in Fig. 6 demonstrating the dominant variations of the mouse shapes. In particular, these refer to bending left, bending right, compressing and stretching for the first two eigenvectors and the third eigenvector encodes more complex variations.

The number of eigenvalues taken into consideration depends on a predefined parameter *f*_*v*_ specifying the variance that contributes to the shape approximation. It is given by the smallest *t* where

13 $$ \sum \limits_{i=1}^{t} \lambda_{i} \geq f_{v} \sum\limits_{i} \lambda_{i}.  $$

The deformable shape model is based on extracting and normalizing the first derivatives **g**_*i*_ of the intensity profiles orthogonal to the contour landmarks. If we assume that **g**_*i*_ is Gaussian distributed, computing the mean profile ${\bar {\mathbf {g}}}$ and the profile covariance matrix **S**_*g*_ allows adapting an unknown shape *g* by minimizing the Mahalanobis distance

14 $$  d_{M}(\mathbf{g_{i}})=(\mathbf{g_{i}}-{\bar{\mathbf{g}}})^{T} \mathbf{S_{g}^{-1}} (\mathbf{g_{i}}-{\bar{\mathbf{g}}})  $$

which is equivalent to maximizing the probability that **g** originates from the Gaussian distribution [27]. The optimal fit along the profile is obtained from an iterative search [29] where the model is shifted and sampled along the normal vector minimizing *d*_*M*_ in Eq. 14. Finally, the model constraints provided by the training set are applied to the updated landmarks [29].

##### Initialization and adaption of the ASM

During mouse interactions, the ASM is positioned and oriented according to the previous frame. Subsequently, a constant number of iterations is alternatingly performed for each ASM in order to adapt segmentation results to the current frame. To avoid that both models merge together, the iterative search along the profiles is restricted to landmarks outside the overlapping area whereas the remaining landmarks are kept in place until the model constraints are applied to the updated landmarks. This strategy on the one hand allows to handle occlusions and on the other hand avoids a gradual attraction of both shapes. The ASM adaption is consequently driven by the landmarks outside the overlapping area where the shape is delineated by clear edges.

**Fig. 7:**
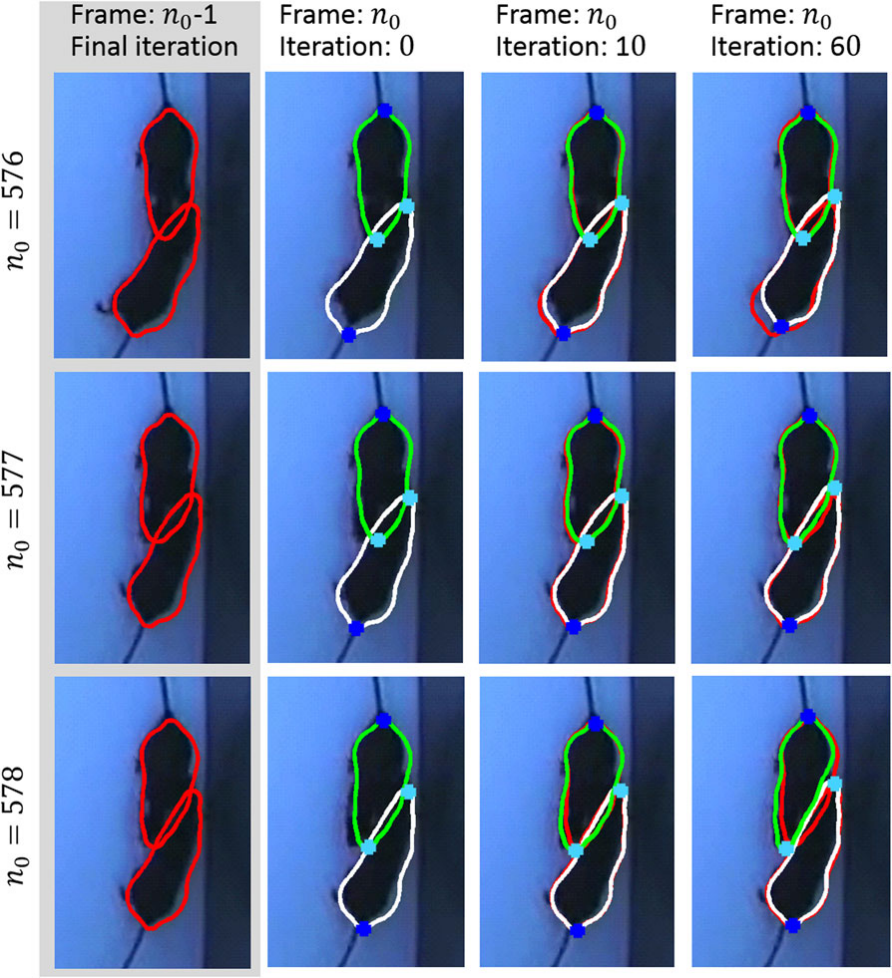
Iteration steps during shape optimization. *First column*: Final segmentation of frame *n*_*o*_−1, *Second* to *fourth column*: next frame *n*_*o*_ and the ASMs after 0, 10 and 60 iterations (*green* and *white contours*)

Exemplarily, the initial segmentations and the results after 10 and 60 iterations for each ASM are shown in Fig. 7 for three successive video frames. Between two consecutive video frames, there is only a slight movement of the animals. Thus, only a limited number of iterations *N*_*max*_ has to be performed for ASM adaption in each frame.

##### Identity preservation

Assigning the correct identity to each mouse is a crucial point for studying social interactions and is a challenge when both mice are close together or partially occluded. Since an ASM is built for each mouse, it keeps track of the identity of an individual during occlusion events. If both mice are spatially separated, the identity is assigned according to the maximum overlap between shapes of successive frames.

## Results

### Parameter settings

**Fig. 8:**
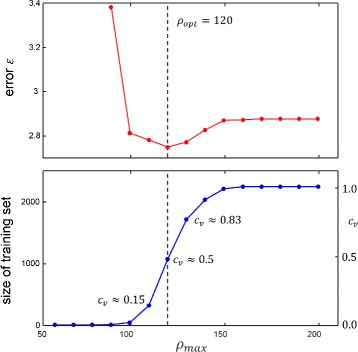
Tracking error *ε* (*top*) and the size of the shape catalog (*bottom*) for different choices of *ρ*_*max*_. The optimum is achieved for *ρ*_*max*_=120 which corresponded to approximately half of the shape candidates (*c*_*v*_=0.5). Evidently, the error changes only marginally around *c*_*v*_= 0.5, so that the influence on the error is assumed to be rather low

One of the most important parameters of the proposed method is the threshold *ρ*_*max*_ directly affecting the size of the shape catalog. It constitutes a trade-off between shape plausibility and variability of the training dataset. If, on the one hand, the threshold is chosen too low, only few variations are learned from the catalog. If, on the other hand, matching costs are too high, the landmarks nose and tail base might not be identified satisfactorily and thus, the training data might not be representative. In order to empirically determine an appropriate value for *ρ*_*max*_, we evaluated the mean error

15 $$ \epsilon = \frac{1}{2} \sum\limits_{f=1}^{N} \left(d_{f}^{Nose,USM} + d_{f}^{Tail,USM} \right)  $$

of nose and tail positions for different values of *ρ*_*max*_ in video V1. The results for *ε* and the corresponding size of the training dataset are shown in Fig. 8. The minimum error is achieved for *ρ*_*max*_=120 where approximately half of the candidate shapes are included into the catalog. As *ρ*_*max*_ depends on the number of frames and landmarks of the ASM, we define the ratio *c*_*v*_ as the number of samples taken for training divided by the total number of samples. According to the experiments shown in Fig. 8, the algorithm performs best if *c*_*v*_ is set to approximately 0.5 meaning that 50% of the shape matchings are used for the shape catalog. Although for *c*_*v*_<0.05 there is a clear decrease in the error rate, within the interval 0.15≤*c*_*v*_≤0.83 the error *ε* changes only marginally in a low subpixel range. The optimization potential for *c*_*v*_ is therefore assumed to be rather low around *c*_*v*_=0.5.

The number of radial and angular bins for the shape matching routine were chosen as proposed by Belongie et al. [25]. Likewise, the ASM was configured with common settings [27] (*m*=3 eigenvalues explaining more than *f*_*v*_=98*%* of the shape variation). The number of iterations, however, should be determined with respect to the sampling rate and the maximum movement of the tracked individual between successive frames. Generally, higher values provide a better adaptivity of the ASM but also involve higher computational costs. In our setup, we considered *N*_*max*_=60 iterations to be more than sufficient for the mice movement.

### Tracking performance

**Fig. 9:**
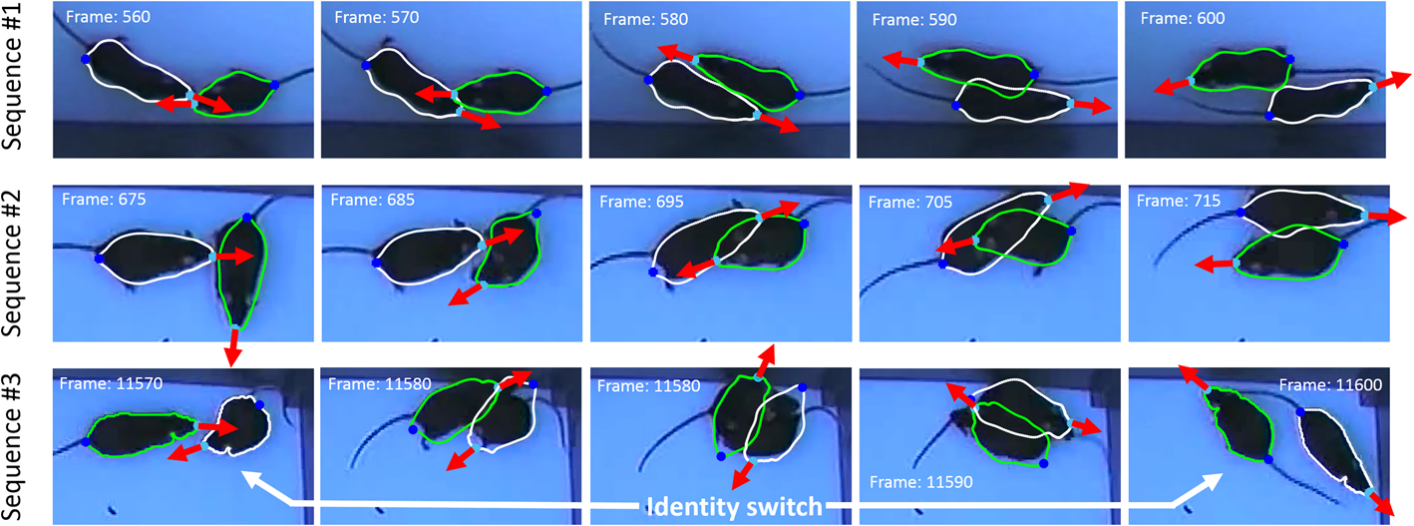
Three different crossing events in video V1. In sequences #1 and #2 the ASM robustly keeps track of both individuals during collision. A switch of the identities occurs in sequence #3

Figure 9 exemplarily illustrates three interactions between both mice taken from video V1. The first and second sequence demonstrate the potential of the unsupervised learning approach even for challenging scenes. Due to several thousand training samples, the ASM shows good agreement with both individuals even dealing with occlusions as illustrated in frame 705 and, moreover, enables to estimate viewing direction during occlusions.

**Fig. 10:**
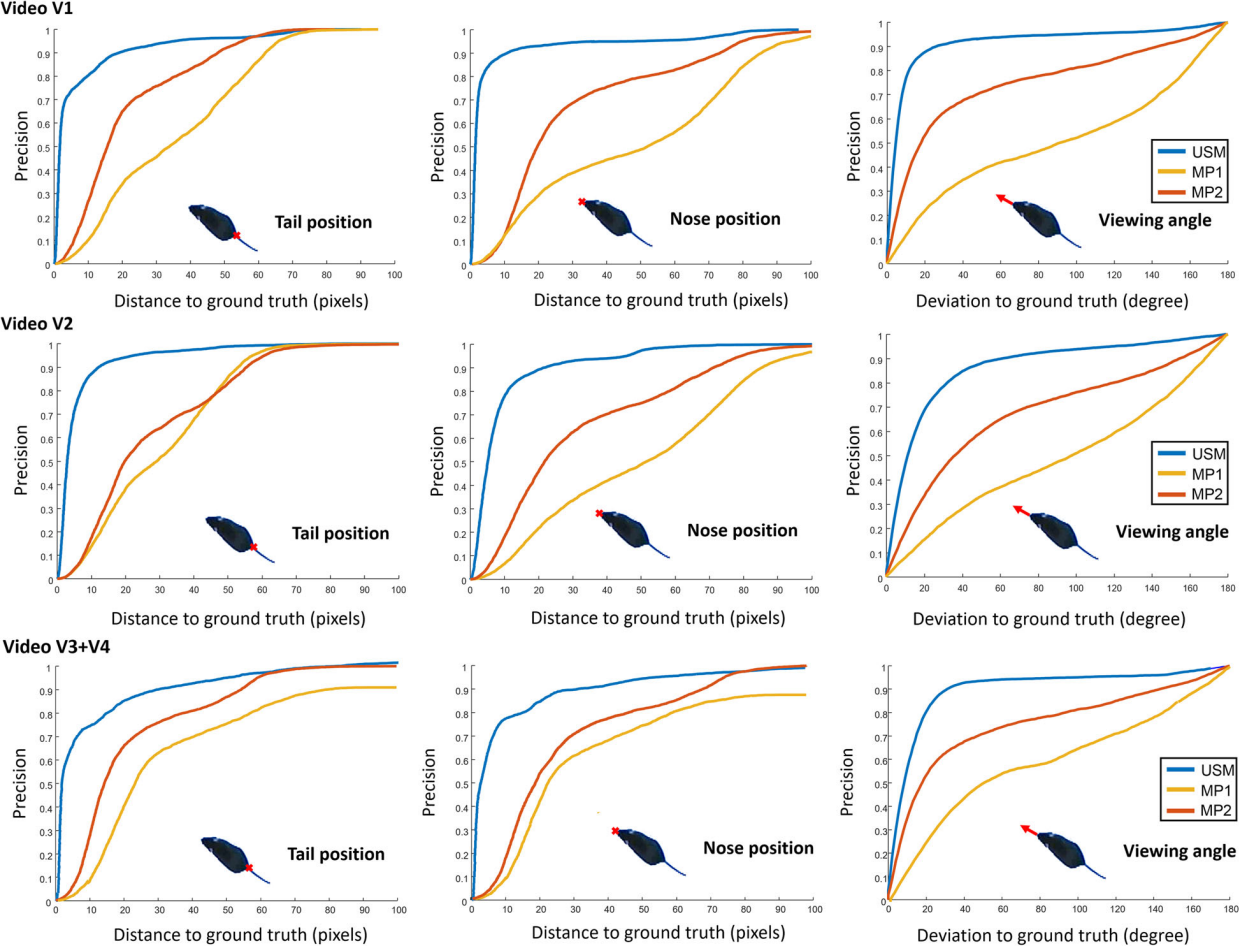
Precision plots showing the tracking accuracy for the tail and nose positions as well as the viewing angle

The tracking performance of the proposed unsupervised learning approach was compared to the MiceProfiler [14, 19]. For this purpose, the MiceProfiler was carefully configured according to the tutorial provided by the authors. We empirically determined binary threshold and mouse model scale parameters that performed best. Due to slightly varying lighting conditions, the threshold had to be adapted during the video to maintain reasonable binary segmentations. Instead of the nose, the physics model implemented in the MiceProfiler software keeps track of the head position. We therefore estimated the optimal extension of the straight line from the shoulder to the head position [19] that minimizes the mean distance to the nose position given in the ground truth. The same strategy was applied for the tail base position by extending the straight line from the belly to the tail position. The viewing angle was extracted from the line connecting shoulder and head positions. In order to evaluate the positional and angular tracking performances of the proposed method and the MiceProfiler, precision plots are shown in Fig. 10 for the estimated nose and tail positions as well as the viewing angle. Precision plots show the percentage of frames (vertical axis) where the deviations of the position or viewing angle is below a given threshold (horizontal axis) from the ground truth [30]. The MiceProfiler was evaluated in two different configurations. In a first setup (MP1), the model has been placed properly at the beginning of the video and was left without interventions until the end. As the authors point out that the MiceProfiler sometimes has problems with contact and overlap, in a second setup (MP2), manual readjustment of both mouse models were performed after each direct interaction. In all precision related evaluations, identity switches were corrected for USM, MP1 and MP2, respectively, and do not affect the precision plots.

The MiceProfiler had considerable problems in keeping the correct orientation, which significantly improved in case of user intervention after interactions. Regarding the open field setup, the optimized contrast brought no improvement in tracking precision for both algorithms. For MP2, precision was even less accurate for the tail base position in the enhanced setting. A clear improvement could be observed for the viewing angle. For USM and MP2, precision increased by approximately 0.2 for deviations of up to 20 degrees. The proposed unsupervised learning scheme clearly outperformed the MiceProfiler in all setups (MP1, MP2) regarding tracking precision of head and tail landmarks as well as the estimated viewing angle.

The number of identity switches occurring for USM, MP1 and MP2 are given in Table 1 for *V*1- *V*4. The proposed algorithm provokes considerably less switches than the MiceProfiler. Likewise, contrast conditions had a major impact on identity preservation for both algorithms, respectively. An example where mouse identities are switched by the USM is illustrated in the third row of Fig. 9. The poor contrast between both mice provokes a rotational shift of the ASMs in frame no. 11580 which continues until mice identities are switched in frame no. 11600.

**Table 1:** Number of identity switches for videos V1 - V4 occurring during the tracking process for USM, MP1 and MP2

	*V*1	*V*2	*V*3	*V*4
Unsupervised approach (USM)	3	1	1	5
MiceProfiler uncorrected (MP1)	16	6	3	14
MiceProfiler corrected (MP2)	12	3	2	12

### Automatic recognition of behavioral states

**Fig. 11:**
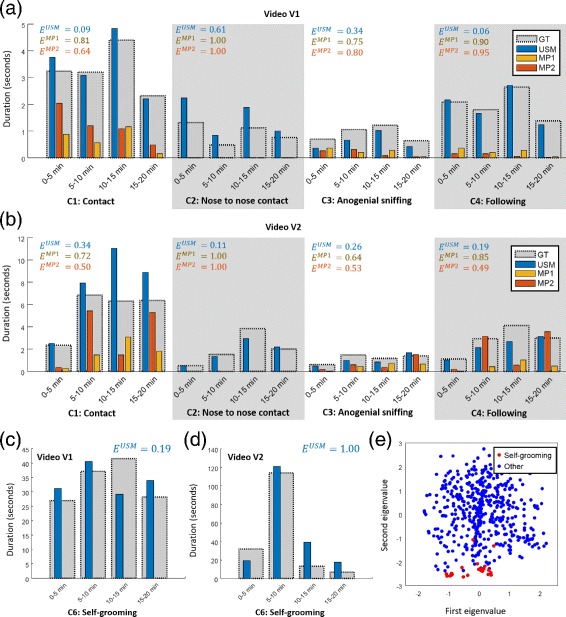
Automated detection of social and non-social interactions. **a**, **b** Duration of social interactions C1–C4 in video (**a**) V1 and (**b**) V2 estimated by the tracking algorithms (USM, MP1, MP2) compared to the ground truth. **c**, **d** Duration of the self-grooming condition C6 in video (**c**) V1 and (**d**) V2 estimated by the tracking algorithms (USM, MP1, MP2) compared to the subjective assessment. **e** PCA space of the first 100 s of mouse no. 1 in video V1 spanned by the first and second eigenvector. Self-grooming conditions are colored in *red*, all remaining samples are *blue*

We compared the automatic behavior classification of the conditions C1-C4 based on the positional and angular data proposed by Chaumont [14] (as described in “Social behavior classification” section) identified by the tracking algorithms (USM,MP1,MP2) and labeled in the ground truth (GT). To evaluate the time evolution of the interactions, we compared the duration of C1-C4 found by the different methods in five minute intervals for both videos (Fig. 11 a and b). The error of duration estimation

16 $$ E^{\{USM,MP1,MP2\}}_{Ci} = \frac{\left| T^{\{USM,MP1,MP2\}}_{Ci} - T^{GT}_{Ci} \right|} {T^{GT}_{Ci} }  $$

was averaged over all time intervals, where $T^{\{USM,MP1,MP2\}}_{Ci}$ denotes the duration of event *C*_*i*_ estimated by the procedure USM, MP1 or MP2 and $T^{GT}_{Ci}$ the duration of *C*_*i*_ derived from the ground truth. Considerable differences between MP and USM were observed for nose to nose and following events. Although nose to nose contact was observed for about 5 s in V1 and 9 s in V2 according to the manually labeled landmarks, it was never recognized by the MiceProfiler (*E* ^*MP*1^=*E* ^*MP*2^=1.0). Likewise, the condition C4: following behavior was rarely recognized by the MiceProfiler in V1 (*E* ^*MP*1^=0.90, *E* ^*MP*2^=0.95). For all categories, a higher accuracy was observed for the USM.

**Fig. 12:**
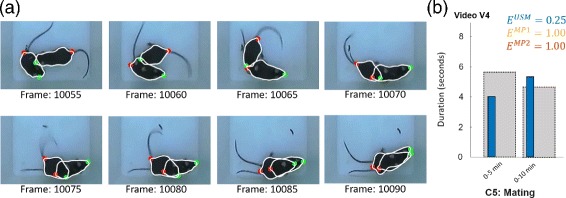
Automatic assessment of the mating condition C5. **a** Sequence involving the mating condition. **b** Duration of the mating condition C5 in video V4 estimated by the tracking algorithms (USM, MP1, MP2) compared to the ground truth

The mating condition C5 was identified for the male-female setup in video V4. Figure 12 exemplarily illustrates the tracking results for the mating condition (Fig. 12 a) as well as the results of the automatic recognition (Fig. 12 b). The video frames demonstrate the challenges for the tracking algorithm. It is remarkable that although there is a high level of occlusion, the ASM works well and delineates the real mice shapes. However, as both ASM are pretty close together, the mating condition is prone to identity switches as shown in Table 1. For the USM, 4 of the 5 switches occur directly after the mating condition. Likewise, the automatic assessment seems to provide a good approximation of the ground truth (*E* ^*USM*^=0.25). In contrast, the MiceProfiler couldn’t cope with such a high level of occlusion and thus, it was not able to recognize condition C5.

The self-grooming condition C6 was identified from the eigenvalue configuration, it was therefore only evaluated for USM. A Support Vector Machine (SVM) was trained in order to identify the duration of the self-grooming condition from the eigenvalues describing the outer boundary of the segmentation. Consistently, validation was performed for each five minute time interval and training from the remaining time of the same video. The SVM was configured with an RBF kernel and was weighted according to the ratio of previously labeled self-grooming to non-grooming conditions in the training set. In video V1, a low error of *E* ^*USM*^=0.19 was achieved, whereas for V2, *E* ^*USM*^=1.00 seems rather error-prone but might be due to the high imbalance of the self-grooming condition C6 over time. Exemplarily, the PCA space of mouse no. 1 for the first 100 s is shown. Grooming conditions are indicated by the red color and non-grooming in blue. Evidently, self-grooming conditions correspond to a low value of the second eigenvalue indicating a stooped body posture (see Fig. 6).

## Discussion

Behavioral screening of manipulated mice is a crucial step for understanding gene function and developing treatments for genetic disorders. In this contribution, we developed an algorithm to automatically track two mice in an enclosed area which makes it possible to automatically assess their social behavior. We implemented a prototype in MATLAB which is not fully optimized yet requiring approximately 4 hours computation time for a 30 min video on a Intel I5 with 3.3 GhZ and 16 GB memory. Despite the comparatively high computational costs, the algorithm is well-suited for large-scale studies due to the accurate tracking results and the low level of necessary user interventions. With respect to tracking accuracy, the number of identity switches and the phenotyping results, the proposed procedure clearly outperforms the recently developed MiceProfiler. Furthermore, due to the iteratively optimization of the ASMs, occlusions can be handled adequately, a feature that is not supported by the MiceProfiler yet. Nevertheless, it has to be noted that the MiceProfiler provides a comfortable solution to assess and to readjust the model landmarks in a frame-by-frame manner and considerably speeds up manual assessments [14].

Model-based tracking approaches often struggle with appearance variations of the scene. Pose variations and shape deformations are among the key challenges for tracking algorithms. In order to tackle these problems, the proposed unsupervised learning algorithm gathers training data during runtime. This has the advantage that appearance variations can be learned from the scene and are thus handled robustly. The procedure showed a high level of robustness even for poor contrast and reflectance conditions. Moreover, the method is able to deal with complex situations during tracking, for example occlusions as illustrated in sequence no. 2 in Fig. 9. As the shape database is built during runtime, the method should principally work with arbitrary species, although parts of the processing pipeline, e.g. the tail detection routine, are specialized for rodent species. Upcoming studies will therefore focus on the method’s generalizability and a more general formulation of the processing pipeline. We expect that the tracker should also be able to cope with insects such as drosophila, ants and various larvae.

An important feature of the proposed method is continuous documentation of shape information during runtime. The eigenvalues reliably indicate self-grooming behavior which is an important non-social parameter showing high relevance e.g. for autism or Huntington’s disease [17, 31]. The high tracking precision of head and tail landmarks, the viewing angle and additional shape information allows an automated and comprehensive assessment of social interactions and non-social behavior. It was demonstrated that behavioral classification was very close to the ground truth which was derived from the manually annotated video frames. Texture descriptors and spatio-temporal features [32] may provide further complementary information for automatic classification and may also increase robustness, which will be considered in future work. We also plan to extend and refine the list of behavioral states as it is not claimed to be exhaustive. For example behavioral conditions such as fighting were not seen in our videos. More complex behavioral states will be addressed in future publications to allow for a more detailed analysis.

Although a high level of tracking precision was achieved, a manual validation of the results is still necessary. It has been observed that, even for optimized contrast conditions, one switch between individuals’ identities occurred during tracking. In this context, the concept of texture-based fingerprints as proposed by Pérez et al. [21] might help to enhance robustness. The procedure does not track individuals, but aims to assign their identities after a successful segmentation. Thus, it could be applied after processing the collisions to correct these switches post-hoc, thereby considerably decreasing the time needed to manually monitor correct identity assignments.

## Conclusion

Mouse models have shown high relevance for understanding genetic and mental diseases and for assessing the efficacy of various therapeutic strategies. A reliable tracking algorithm that requires only minimum user intervention is a crucial prerequisite for any high-throughput behavioral analysis. In this paper, we propose an unsupervised learning procedure which copes with direct mouse interactions, occlusions and poor contrast conditions. As training data is gathered during runtime, only minimal user input is required to initiate the tracking process. The proposed method was found to track head and tail landmarks precisely and, furthermore, enables identification of non-social conditions such as self-grooming which is a crucial parameter for several mice models. Overall, the proposed method shows substantial potential to automate behavioral screening of mice and other animals.

## Abbreviations

ASM: Active shape model
RFID: Radio-frequency identification
